# Comparison of matrix metalloproteinase 9 and 14 levels in vitreous samples in diabetic and non-diabetic patients: a case control study

**DOI:** 10.1186/s40942-022-00394-0

**Published:** 2022-06-21

**Authors:** A. Ünal, O. Baykal, N. Öztürk

**Affiliations:** 1 Faculty of Medicine, Department of Ophthalmology, Artvin State Hospital, Atatürk University, Çarşı District, Hospital Street, No:5, 08000 Artvin, Turkey; 2 Faculty of Medicine, Department of Ophthalmology, Ataturk University, Erzurum, Turkey; 3 Faculty of Medicine, Department of Biochemistry, Atatürk University, Erzurum, Turkey

**Keywords:** Matrix metalloproteinase, Diabetic retinopathy, Pars plana vitrectomy

## Abstract

**Background:**

MMP-9 plays a prominent role in inflammation and MMP-14 take part in angiogenesis. The objective of this study is to compare MMP-9 and MMP-14 levels between diabetic and non-diabetic patients.

**Methods:**

The patients who scheduled for pars plana vitrectomy were included in our study. Patients are divided into 2 groups: the diabetic group and non-diabetic group. Age, gender, intraocular pressure(IOP), visual acuity (VA) were reported. Color fundus photography, fundus fluorescein angiography, optic coherence tomography (OCT) were performed before and after the operation. MMP-9 and MMP-14 levels in vitreous samples were analyzed with a reader device by ELISA method. Mann–Whitney *U* test and logistic regressions were used in statistical analysis, p < 0.05 accepted as statistically significant.

**Results:**

70 eyes of 70 patients who received pars plana vitrectomy were enrolled in the study and divided into 2 groups: 34 patients in the diabetic group, 36 patients in the non-diabetic group. The average age of diabetic patients was 60.14 ± 10.20, and non-diabetic patients was 64.22 ± 11.16, respectively. The average MMP-9 (0.67 ± 0.66 ng/ml) and MMP-14 (0.16 ± 0.45 ng/ml) values in the diabetic group were significantly higher than the average MMP-9 (0.21 ± 0.05 ng/ml) and MMP-14 (and 0.07 ± 0.02 ng/ml) values in the non-diabetic group (P < 0.01). Also, it was observed that MMP-9 and MMP-14 levels increases as the diabetic disease duration increases. The risk of diabetes incidence increased with high levels of MMP-9 and MMP-14.

**Conclusion:**

Due to the higher levels of MMP-9 and MMP-14 in the pathogenesis of diabetic retinopathy, these proteins may probably be among the therapeutic targets in the prevention and treatment of retinopathy.

## Introduction

Diabetic retinopathy which is progressive and gradually damages the retinal vessels is one of the most common ocular findings in diabetic patients. This condition is asymptomatic in the initial stages of diabetes, but if diabetes is not treated, it may even result in blindness [1]. Diabetic retinopathy is a pathological and complex disease which causes retinal ischemia, increased retinal permeability, retinal neovascularization, macular edema, and microvascular changes [2, 3].

In the treatment of diabetic retinopathy, interventions aiming treatment of diabetes are sometimes insufficient. Thus, alternative treatments are required. The role of molecular mechanisms in the development of diabetic retinopathy should be explained and therapeutic goals might be set [4].

Matrix metalloproteinase (MMP) family is a zinc-dependent proteinase family with more than 25 members. MMPs family regulates the structure of the extracellular matrix in physiological and pathological processes [5]. It has a central role in organ development and tissue remodeling. Activation of MMPs accelerates apoptosis by a process involving mitochondria through various factors [6]. The MMPs family has a dual role in the development of diabetic retinopathy; It accelerates the apoptosis of retinal capillary cells, pericytes the microvessel regulation and disrupts endothelial cells in the early stages of diabetic retinopathy and it supports the formation of new vessels in the neovascular stage [6, 7].

MMP-9 is the largest and most complex molecule of the MMPs family [8]. The MMPs family regulates the physiological and pathological processes of the subcellular and intracellular region according to its location. MMP-9 is also involved in many inflammatory events with release activation in latent form [9]. MMP-9 is produced through very different stages such as gene transcription, synthesis, secretion, activation, inhibition and glycosylation [10]. In a recent study, MMP-9 gene modified rats were made diabetic and their retinas were examined. Rapid loss of retinal capillary cells was prevented and retinopathy in the early stages of diabetes was revealed [11].

Also, MMP-14, a transcollagenous protein, or type 1 membrane of MMPs family, has an important and critical role in the communication of cells with each other and in the remodeling and invasion of the cells into the extracellular matrix. MMP-14 has direct activity against some of the extracellular matrix proteins, such as gelatin, fibronectin, vitronectin, fibrillar collagen, and aggregates [12]. Studies have shown that MMP-14 plays a very critical and important role in angiogenesis [13]. MMP-14 is found in higher concentrations in many tumor tissues and is involved in tumor progression, angiogenesis, and metastasis [14, 15]. For example, In Kaimal et al.'s study of mice with ovarian cancer, MMP-14 was increased in the serum of women with malignant ovarian tumors.

MMPs pharmacological inhibitors make the inhibition by blocking the zinc-dependent site. Doxycycline is a broad spectrum MMP inhibitor that inhibits many MMPs, including MMP-9 [16]. Another inhibitor, bisphosphonates, make MMP inhibition by chelating with the zinc cation site [17]. Because MMP-9 is closely associated with inflammation, non-steroidal anti-inflammatory drugs, such as indomethacin, reduce prostaglandin E2 synthesis and MMP-9 levels and Trastuzumab reduce MMP-9 as confirmed in TOGA trial study [18, 19]. Pharmacological inhibition of MMPs in the development of diabetic retinopathy protects from retinal and choroidal neovascularization [20] and prevents MMP-9-related vascular permeability and inflammation [21]. Also, the use of synthetic MMP inhibitors prevents the induction of proliferative vitreoretinopathy [22]. Inhibition of MMP-9 with COX inhibitors prevents retinal pathologies [23].

Studies about MMP inhibitors and treatment in literature mostly relate to animal models or other in vitro studies, and not to humans. Inhibition of the MMP-9 and MMP-14 proteins may be an alternative for suppressing diabetic retinopathy. Thus, we aimed to compare MMP-9 and MMP-14 levels in diabetic and non-diabetic patients which could be the biomarkers of diabetic retinopathy.

## Methodology

70 eyes of 70 patients who applied to Atatürk University Faculty of Medicine Ophthalmology Outpatient Clinic with the complaint of visual impairment and were scheduled for surgery with vitreoretinal pathology in the ophthalmological examination were included in our study. A systematic sample size calculation was not done. With the purposeful sampling, all patients who were to be operated on due to the problems mentioned in the study and who gave consent for the study in a 1-year period were included in the sample. All patients in the diabetic group were in the proliferative diabetic retinopathy stage and unresponsive to medical treatment. The patients were recruited based on diabetic status: the diabetic group and non-diabetic group. The study was conducted with the approval of the Ethics Committee of Atatürk University Faculty of Medicine, Clinical Research Ethics Committee, numbered B.30.2.ATA.0.01.00/335. Before the study, the patients were given detailed information about the purpose of the study and the procedures to be performed. The Helsinki declaration was followed and informed consent forms were obtained from the patients.

The pathologies requiring vitreoretinal surgery were grouped within themselves. Detailed medical history was taken from the patients. In the study group for whom vitreoretinal surgery was planned, patients who had received anti-VEGF treatment due to an ocular disease or had any previous ocular surgery and those who used medication for ocular disease were excluded from the study. Patients with pathology in both eyes requiring vitreoretinal surgery were also excluded from the study. The patients in the control group underwent vitreoretinal surgery because of the macular hole (n = 20) and epiretinal membrane (n = 16).

Best corrected visual acuity (BCVA), intraocular pressure (IOP) measurement with applanation tonometry (Haag Streit, Bern, Switzerland), anterior segment examination and fundus examination were performed before and after the operation. Also, HbA1c levels of the patients were measured.

Vitreous material collection was taken under appropriate operating conditions, under general or local anesthesia, during pars plana vitrectomy surgery. After conjunctival incisions made in the lower nasal and upper temporal regions with scissors, 3 cc subtenon anesthetic substance (2 cc Lidocaine and 1 cc Bupivacaine) was applied to all patients undergoing local anesthesia. The conjunctival sac was washed with povidone iodine 5%. Transsclerotomy was performed from the lower temporal, upper temporal and upper nasal 3–4 mm behind the surgical limbus and entered as a pars plana. At this stage, 0.3–0.6 ml of vitreous was taken by vitreous tap method using a vitrectomy cutter just before the infusion cannula was inserted [24]. Then, the vitreoretinal surgery was completed by entering the infusion cannula, light probe and vitrectomy probe. Silicone oil, gas material (C3F8), medical air or balanced salt solution were used as the tamponade. Vitreous samples taken were placed in Eppendorf tubes under sterile conditions. Tubes were frozen at − 80° C without waiting to preserve the materials.

MMP-9 and MMP-14 levels in vitreous samples are taken into its special apparatus Sunlong ([Cat No: SL1157Hu for MMP-9, Cat No: SL2924Hu for MMP-14, Sunglong Biotech Co. Ltd, Zhejiang, China]) according to the standard protocol suggested by the manufacturer, Dynex brand automatic ELISA (Enzyme linked Immunosorbent Assay) was analyzed with a reader device (Dynex Technologies Headquarters, Chantilly, USA) by ELISA method. The pre-measurement dilution step recommended in the kit measurement protocol was not performed because the levels of these molecules in vitreous samples were expected to be low. The intra-assay coefficient of variation (CV) value of the kit was below 10% and the inter-assay CV below 12%.

### Statistical analysis

Statistical analysis was performed using the SPSS 22.0 (SPSS, Chicago IL, United States) program. Because the study sample was small, the non-parametric Mann–Whitney *U* test was applied to define whether there is a significant difference of MMP-9 and MMP-14 between the diabetic and non-diabetic groups. P value ≤ 0.05 was considered to indicate statistical significance.

## Results

A total of 70 patients who received pars plana vitrectomy were enrolled in the study and divided into 2 groups: 34 patients in the diabetic group, 36 patients in the non-diabetic group. Table 1 presents the gender and age distributions of the patients. 19 (55.9%) of the diabetic patients were male and 15 (44.1%) were female. The patients in the non-diabetic group consisted of 15 (41.7%) men and 21 (58.3%) women. The mean age of diabetic patients was 60.14 ± 10.20, while the mean age of non-diabetic patients was 64.22 ± 11.16. (Table 1).

**Table 1:** Demographic, clinic and laboratuary ınformations of patients

	Diabetic (n = 34)	Non-diabetic (n = 36)
Gender		
Women	15 (44.1%)	21 (58.3%)
Men	19 (55.9%)	15 (41.7%)
Age	60.14 ± 10.20 (38–77)	64.22 ± 11.16 (33–81)
HbA1c level	7.5059 ± 0.727,778	5.138,889 ± 0.322,736
Vitrectomy ındications		
Tractional retinal detachment	16 (47.1%)	–
Vitreous hemorrhage	18 (52.9%)	–
Macular hole	–	20 (55.5%)
Epiretinal membrane	–	16 (44.4%)

The average HbA1c levels were 7.5059 ± 0.727,778 for diabetic group and 5.138,889 ± 0.322,736 for non-diabetic group. The patients in diabetic group were undergone vitreoretinal surgery for tractional retinal detechment (n = 16) and vitreous hemorrahage (n = 18). Vitrectomy indications of non-diabetic group were macular hole (n = 20) and epiretinal membrane (n = 16) (Table 1).

**Fig. 1:**
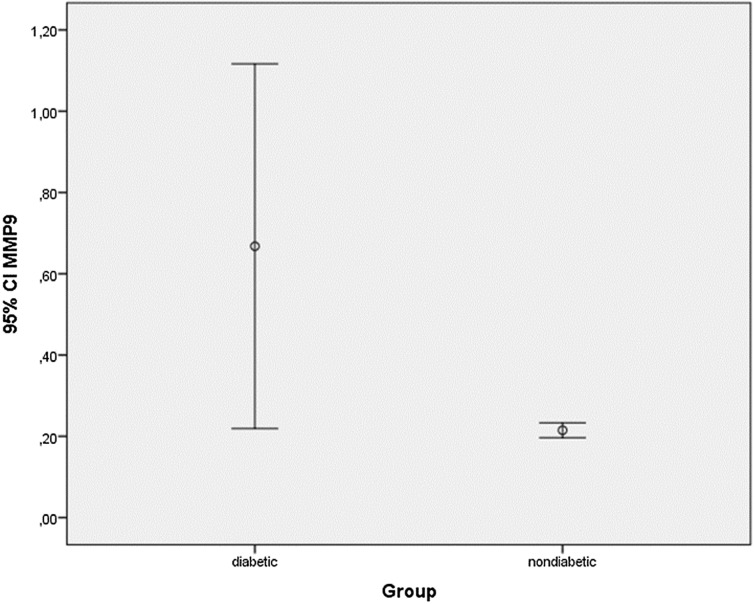
Comparison of MMP-9 levels in diabetic/nondiabetic groups

**Fig. 2:**
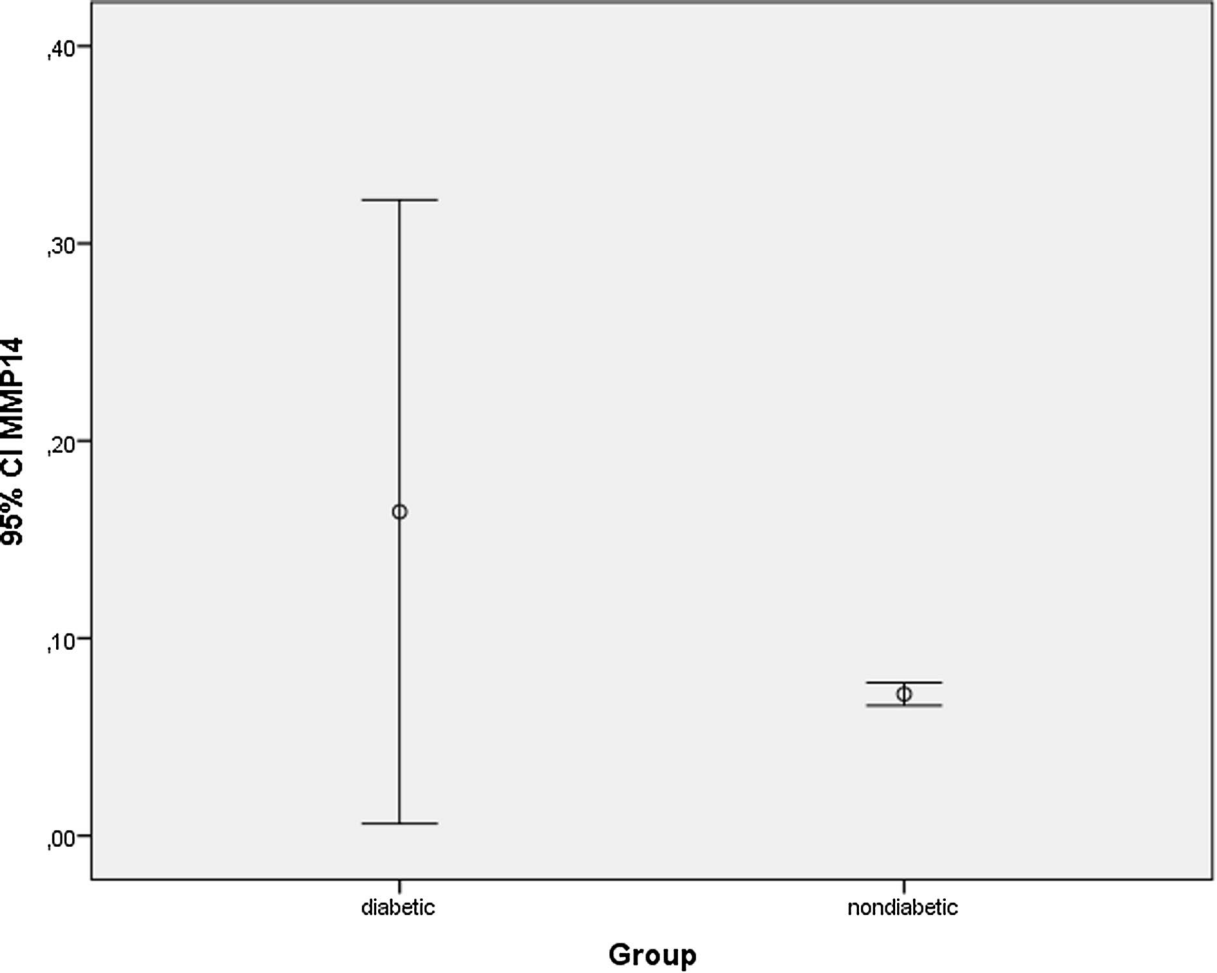
Comparison of MMP-14 levels in diabetic/nondiabetic groups

A significant difference was found between diabetic and non-diabetic groups in terms of MMP-9 level (*U* = 363.500; *p* = 0.003) and MMP-14 level (*U* = 404.500; *p* = 0.015). MMP-9 and MMP-14 values of the patients in the diabetic group were significantly higher than the non-diabetic group (*p* < 0.01)*.* The average (SD) MMP-9 value *x̄* = 0.6677 (± 1.28602) in diabetic group was significantly higher than the average (SD) MMP-9 value *x̄* = 0.2149 (± 0.05381) in non-diabetic group. Also, the average (SD) MMP-14 value *x̄* = 0.1641 (± 0.45261) in diabetic group was significantly higher than the average (SD) MMP-14 value *x̄* = 0.0718 (± 0.01718) in non-diabetic group. There was no significant difference between Tractional Retinal Detachment and Vitreous Hemorrhage as diabetic subgroups in terms of MMP-9 and MMP-14 levels p > 0.05 (Table 2). Distribution of MMP-9 and MMP-14 values in diabetic and nondiabetic groups are shown in Figs. 1 and 2, respectively.

**Table 2:** Comparison of MMP-9 and MMP-14 levels of diabetic and non-diabetic groups

	Diabetic (Mean ± Std. Dev) (ng/ml)		Non-diabetic (Mean ± Std. Dev) (ng/ml)	Mann–Whitney U	Z	*p* value	Effect size (95% CI)
MMP-9	(0.668 ± 1.28)		(0.2149 ± 0.053)	363.500	− 2.922	0.003*	(0.025–0.881)
MMP-14	(0.1641 ± 0.453)		(0.0718 ± 0.017)	404.500	− 2.439	0.015*	(− 0.058–0.243)

	Tractional retinal detachment	Vitreous hemorrhage		Mann–whitney U	Z	*p* value	Effect size (95% CI)
MMP-9	(0.4205 ± 0.315)	(0.8629 ± 1.692)		117.000	− 0.885	0.391	(− 1.347–0.462)
MMP-14	(0.0894 ± 0.035)	(0.2231 ± 0.605)		141.000	− 0.052	0.973	(− 0.453–0.186)

According to Table 3, a significant positive correlation was found between HbA1c levels and MMP-9 and MMP-14 levels of patients. While HbA1c levels increase, the MMP-9 (*r* = 0.661, *p* = 0.000) and MMP-14 (*r* = 0.468, *p* = 0.005) levels also increase.

**Table 3:** Correlation between HbA1c and MMP-9 and MMP-14 levels in diabetic group

	HbA1c	MMP-9	MMP-14
HbA1c	1	0.661	0.468
MMP-9	0.661	1	0.960
MMP-14	0.468		1

When the correlation between diabetes duration and MMP-9 and MMP-14 levels was examined, it was observed that the level of these proteins increased significantly as the disease duration increased (p < 0.05). (Table 4).

**Table 4:** Correlation between the duration of the diabetes and MMP-9 and MMP-14 Levels

	MMP-9	MMP-14
Duration	0.665**	0.518

Binary logistic regression analysis was performed to determine the risk factors of the presence of diabetes and the effect of MMP-9 and MMP-14 levels on being diabetic/nondiabetic group. Age and gender variables also were included in the analysis. The risk of diabetes incidence was found to increase in cases of high MMP-9 (*β* = − 5.853, *p* = 0.006) and high MMP-14 (*β* = − 31.613, *p* = 0.18).

## Discussion

We compared MMP-9 and MMP-14 levels in vitreous between diabetic and non-diabetic groups to analyze novel markers of diabetic retinopathy as diabetes is a risk factor of diabetic retinopathty. Diabetic retinopathy is a systemic and epidemic disease affecting millions of people worldwide [25].

Current treatment includes insulin therapy in Type 1 diabetes and single or multiple combinations of sulfanylurea, diazolidediones, biguanides, alpha glucosidase inhibitors, meglitinides and insulin in Type 2 diabetes [26]. However, many of these drugs may not treat the microvascular mechanisms that cause diabetic retinopathy. Hyperglycemic vascular stress persists even if blood glucose is brought within normal limits. The main reasons are the advanced glycation end products [27], mitochondrial damage and oxidative stress [28], the epigenetic changes that have occurred [28, 29].

Detected hyperglycemia is seemed to be the main determinant in diabetic retinopathy. However, it is not clear how hyperglycemia produces retinal pathology. In the pathogenesis of diabetic retinopathy, an accelerated apoptosis process is observed in retinal cells, capillary cells, müller cells, and ganglion cells [30]. Apoptosis in capillary cells leads to characteristic microvascular pathologies in diabetic retinopathy. Accelerated apoptosis also expresses how the destruction of pericyte cells occurs [31]. Blood pressure control and blood sugar regulation are sometimes not possible and adjunctive treatments are required. Thus, molecular mechanisms in the development of diabetic retinopathy should be investigated [4].

MMPs family has a central role in organ development and tissue remodeling. Activation of MMPs accelerates apoptosis by a process involving mitochondria through various factors [6]. MMPs activation has been shown to accelerate the apopitosis process in some studies. MMP-9 activity affects retinal cells and their function in the eyes. MMP activity or physiological disturbances can lead to disruption of the ECM, resulting in ocular damage and malfunction of various cell types in the eyes. The MMPs family has a dual role in the development of diabetic retinopathy; It accelerates the apoptosis of retinal capillary cells, pericytes the microvessel regulation and disrupts endothelial cells in the early stages of diabetic retinopathy and it supports the formation of new vessels in the neovascular stage [6, 7].

In recent studies, MMP-9 gene modified rats which were made diabetic and their retinas were examined, rapid loss of retinal capillary cells was prevented and retinopathy in the early stages of diabetes was revealed [11]. Studies have shown that MMP-14 plays a very critical and important role in angiogenesis [13]. MMP-14 is found in higher concentrations in many tumor tissues and is involved in tumor progression, angiogenesis, and metastasis [14, 15].

MMPs pharmacological inhibitors make the inhibition by blocking the zinc-dependent site. Doxycycline is a broad spectrum MMP inhibitor that inhibits many MMPs, including MMP-9 [16]. Another inhibitor, bisphosphonates, make MMP inhibition by chelating with the zinc cation site [17]. Because MMP-9 is closely associated with inflammation, non-steroidal anti-inflammatory drugs, such as indomethacin, reduce prostaglandin E2 synthesis and MMP-9 levels [32]. Pharmacological inhibition of MMPs in the development of diabetic retinopathy protects from retinal and choroidal neovascularization [7] and prevents MMP-9-related vascular permeability and inflammation [33]. Also, the use of synthetic MMP inhibitors prevents the induction of proliferative vitreoretinopathy [22]. Inhibition of MMP-9 and MMP-2 with COX inhibitors prevents retinal pathologies [23], but there is no clinical study on the treatment of diabetic retinopathy patients with MMP inhibitors.

In the treatment of diabetic retinopathy, interventions aiming treatment of diabetes are sometimes insufficient. Thus, alternative treatments are required. Inhibition of the MMP-9 and MMP-14 proteins may be an alternative for suppressing diabetic retinopathy. Many different protein levels in the vitreous can be examined to the extent which technology allows. The MMPs family is a protein found in many regions of the body at crucial stages in wound remodeling, inflammation, angiogenesis, and tumor tissues. In the expert opinion presented by Kowluru et al. based on their literature review, MMP-14 is recommended as a biomarker in the treatment of diabetic retinopathy. MMP-9 is often found elevated levels in inflammation, autoimmune degenerative diseases and conditions requiring angiogenesis [34].

In some studies, it has been found that the level of MMPs in diabetic retinopathy and proliferative diabetic retinopaty is higher than the non-diabetic vitreous [35, 36]. The findings of our study are consistent with findings of studies of El-Asrar et al. [24, 35] and Jin et al. [36] in which MMP-9 levels were higher in diabetic patients than non-diabetic patients. In the study of Abu El-Esrar et al., MMP-14 is recommended as a bio-marker, MMP-9 is additionally recommended in our study.

In our study, similar to the study of Giebel et al. [6] in bioequivalent rats samples, MMP-14 level was found to be significantly higher in diabetic group compared to the non-diabetic group in human samples. Kowluru et al. [37] in 2014 showed that the increase in MMP-9 level in diabetes increased oxidative stress in retinal capillary cells via mitochondria and consequently accelerated apoptosis in retinal capillary cells. Retinal mitochondrial hemostasis continues normally in rats blocked the MMP-9. It has been shown that the development of diabetic retinopathy in these rats was inhibited. Also, regulation of oxidative stress by pharmacological/genetic approaches maintains retinal mitochondrial homeostasis in diabetic rats by improving epigenetic modifications in the MMP-9 promoter region in a study [38].

Our results showed that MMP-9 and MMP-14 enzymes were significantly higher in diabetic patients compared to nondiabetic patients. Also, it was observed that MMP-9 and MMP-14 levels increases as the disease duration increases. However, no difference was found between different diabetic medical conditions. The risk of diabetes incidence increased with high levels of MMP-9 and MMP-14. Also, we found a positive correlation between HbA1c levels and MMP-9 and MMP-14 levels in diabetic group. This result supports that MMP-9 and MMP-14 could be a biomarkers of diabetic retinopathy. Diabetic retinopathy can be suppressed by inhibition of MMP-9 and MMP-14 levels which indicates a new form of treatment. MMP-9 has a very important role in diabetic retinopathy and its increased levels are one of the main factors causing retinal capillary apoptosis. Blocking the cascade at any stage prevents the effects of diabetic retinopathy in theoretical and experimental terms. Jayashree et al., found higher serum MMP-9 levels in Diabetes Mellitus with type 2 patients with retinopathy, as compared with those without retinopathy. While we found high levels of MMP-9 in the vitreous samples of diabetic patients in our study, these researchers found high serum MMP-9 levels in diabetic rats. They conclude that rise in MMP-9, and associated serum markers promote disease progress in DR [39]. In an experimental study by Bhatt and Addepalli, the combination of minocycline and aspirin inhibited MMP-2 and MMP-9, and improved retinal thickness and blood-retinal barrier in diabetic mice. Authors suggest the beneficial effects of treatment with this combination in diabetic neuropathy [23]. Thus, treatments targeting the inhibition of MMP-9 and MMP-14 can be used in the treatment of diabetic retinopathy. Experimental studies with the rats mentioned above, while the first study found the higher serum MMP-9 level, the second study determined the MMP-9 levels by histological examination of the retinal section. Although the common diabetes context, the fact that our study was carried out with human vitreous samples constitutes its distinctive feature. At this point, this study constitutes an important pillar in the efforts to create a biomarker in the treatment of diabetic retinopathy.

There are few studies in the literature examining the relationships between diabetic retinopathy and MMP’s family. Also, most of these studies used bioequivalent living vitreous instead of human vitreous. The sample size in this study is higher than in the few studies conducted with patients. While some of the previous studies emphasized MMP-9 or MMP-14, both were analyzed in this study. Also, both MMP-9 and MMP-14 levels were found significantly higher in the diabetic group compared to the non-diabetic group. In the analyses, the effects of some variables such as age and gender were controlled. It was also investigated whether there was a difference in MMP-9 and MMP-14 levels in terms of two disease categories in the diabetic group. These proteins may probably be among the therapeutic targets in the prevention and treatment of diabetic retinopathy.

Following researches involving experimental interventions, levels of MMP-9 and MMP-14 can be used in the diagnosis and assessment of the severity and in treatments that can reduce or slow eye damage caused by diabetic retinopathy by suppressing the level of these proteins that represents the clinical contribution of this study. High MMP-9 and MMP-14 levels are associated with rapid progression of diabetes which can be considered as prognostic factors. Drugs targeting this protein can be used to slow or stop the progression of diabetes that may be useful for clinicans (see Table 5).

**Table 5:** Logistic regression analysis for diabetic/nondiabetic

Step	Variables	*β*	Standart error	Wald	*p* value	Exp(β)	95% CI for OR	
1	Gender	− 0.506	0.608	0.693	0.405	0.603	0.183	1.985
	Age	0.056	0.029	3.752	0.053	1.057	0.999	1.119
	MMP-9	− 5.839	2.190	7.111	0.008	0.003	0.000	0.213
	MMP-14	− 32.949	13.592	5.876	0.015	0.000	0.000	0.002
	Constant	1.156	1.905	0.369	0.544	3.178		
2	Age	0.052	0.028	3.361	0.067	1.053	0.996	1.113
	MMP-9	− 5.853	2.138	7.495	0.006	0.003	0.000	0.190
	MMP-14	− 31.613	13.365	5.595	0.018	0.000	0.000	0.004
	Constant	1.054	1.892	0.310	0.578	2.868		

## Limitations

Other systemic and local diseases that could affect these protein levels were excluded from the study. Thus, we believe the sample size is sufficient to obtain these results. Patients in the nondiabetic group in this study have different diseases. However, these levels could only be measured after vitroretinal surgery which is operated in some pathological conditions. It is not possible to make these measurements with completely healthy individuals.

## Conclusion

In conclusion, this study showed that the diabetic retionapathy have increased MMP-9 and MMP-14 protein levels. These proteins could be the biomarkers of diabetic retinopathy. Thus, MMP-9 and MMP-14 protein levels can be used for the diagnosis and treatment in diabetic retinopathy. In addition to diabetes treatments, MMP-9 and MMP-14 inhibitors can be used to slow down the progression of diabetic retinopathy and to prevent retinal damage. These results should be confirmed by larger studies.

## Data Availability

The data of this study are available on request from the corresponding author. The data are not publicly available due to restrictions that could compromise the privacy of research participants.

## Abbreviations

MMP: Matrix metalloproteinase
anti-VEGF treatment: Anti–vascular endothelial growth factor treatment
BCVA: Best corrected visual acuity
IOP: Intraocular pressure
HbA1c levels: Hemoglobin A1c levels
